# Early pathological mechanisms in a mouse model of heart failure with preserved ejection fraction

**DOI:** 10.1152/ajpheart.00318.2024

**Published:** 2024-11-01

**Authors:** Paola C. Rosas, Liomar A. A. Neves, Nisha Patel, Duyen Tran, Carlos H. Pereira, Karina R. Bonilla, Jingjing Zheng, Jun Sun, Francisco J. Alvarado, Kathrin Banach

**Affiliations:** ^1^Department of Pharmacy Practice, College of Pharmacy, University of Illinois at Chicago, Chicago, Illinois, United States; ^2^AbbVie Inc, Chicago, Illinois, United States; ^3^Department of Internal Medicine/Cardiology, Rush University Medical Center, Chicago, Illinois, United States; ^4^Department of Medicine and Cardiovascular Research Center, University of Wisconsin-Madison School of Medicine and Public Health, Madison, Wisconsin, United States; ^5^Division of Gastroenterology and Hepatology, Department of Medicine, University of Illinois at Chicago, Chicago, Illinois, United States; ^6^UIC Cancer Center, University of Illinois at Chicago, Chicago, Illinois, United States

**Keywords:** HFpEF, inflammation, intestinal atrophy, mineralocorticoid, western diet

## Abstract

Heart failure with preserved ejection fraction (HFpEF) constitutes more than half of all HF cases, yet evidence-based therapies remain lacking due to limited understanding of its underlying pathological mechanisms. Our study aimed to uncover early pathological mechanisms in HFpEF by exposing mice to dietary conditions resembling a Western diet—rich in fats, salt, and low in fiber—alongside excess mineralocorticoids to replicate significant aspects of human HFpEF. Echocardiography was performed at both 3-wk and 6-wk intervals postchallenge, revealing cardiac alterations as early as 3 wk. While ejection fraction remained preserved, mice exhibited signs of diastolic dysfunction, reduced stroke volume, and left atrial enlargement. In addition, changes in pulmonary flow velocities were noted by the 3-wk mark, suggesting elevated pulmonary pressure. Extracardiac comorbidities included organ congestion, increased adiposity, impaired glucose tolerance, and hypercholesterolemia. Molecular analyses unveiled evidence of low-grade inflammation, oxidative stress, and impaired NO-cGMP-PKG signaling, contributing to the observed decrease in titin phosphorylation, thereby impacting myocardial stiffness. In addition, impaired nitric oxide (NO) signaling might have influenced the alterations observed in coronary flow reserve. Moreover, dysregulation of calcium signaling in cardiomyocytes and reduced sarcoplasmic reticulum (SR) load were observed. Interestingly, elevated phosphorylation of cMyBP-C was linked to preserved ejection fraction despite reduced SR load. We also observed intestinal atrophy, possibly due to a high-fat diet, low dietary fiber intake, and diminished gut perfusion, potentially contributing to systemic low-grade inflammation. These findings reveal how excess mineralocorticoid salt-induced hypertension and dietary factors, like high-fat and low-fiber intake, contribute to cardiac dysfunction and metabolic disturbances, offering insights into early HFpEF pathology in this model.

**NEW & NOTEWORTHY** Our study demonstrates that feeding mice a Western diet rich in fat and salt and low in fiber alongside excess mineralocorticoids replicates aspects of human HFpEF. Cardiac alterations including diastolic dysfunction and decreased stroke volume with preserved ejection fraction were observed. Extracardiac effects included organ congestion, adiposity, glucose intolerance, and intestinal atrophy. Molecular analysis revealed inflammation, oxidative stress, impaired NO-cGMP-PKG signaling pathways, and altered calcium signaling in cardiomyocytes, shedding light on early pathological changes in HFpEF.

## INTRODUCTION

Heart failure with preserved ejection fraction (HFpEF) represents a significant clinical challenge (1, 2), characterized by diastolic dysfunction, preserved ejection fraction, and associated comorbidities such as obesity, hypertension, and diabetes (3). Despite its increasing prevalence and substantial morbidity and mortality rates, effective therapeutic strategies for HFpEF remain elusive. Most drugs effective for heart failure with reduced ejection fraction (HFrEF) have not shown the same effectiveness for HFpEF. Some drugs, like the mineralocorticoid receptor antagonist (MRA) spironolactone (4), the angiotensin receptor antagonist coupled with neprilysin inhibitor (ARNI) sacubitril/valsartan (5), and the sodium-glucose cotransporter-2 (SGLT2) inhibitors empagliflozin (6) and dapagliflozin (7), have shown some effectiveness for treating HFpEF. However, we still lack a clear understanding of how these drugs provide cardioprotection. Understanding of the pathophysiological mechanisms driving HFpEF is crucial for developing novel targeted therapies to improve outcomes in affected patients.

Advancement in our understanding and treatment of HFpEF has been hindered by limitations in preclinical animal models, which fail to fully represent the complexity and multiorgan nature of this disease. Thus, using different models may be necessary to encompass the clinically heterogeneous range of HFpEF phenotypes observed in humans (8). Hypertension, diabetes, and obesity are among the most prevalent factors associated with HFpEF, alongside numerous cardiac and noncardiac comorbidities. Animal models that use multiple challenges to replicate the comorbidities of HFpEF may offer a more accurate representation of the disease compared with models using a single organ challenge approach (9–12). In our study, we used a novel approach to induce HFpEF by combining excess mineralocorticoid with increased salt intake, previously used to induce hypertension (13, 14), along with a high-fat, low-fiber diet to induce metabolic syndrome. This approach aims to replicate the most prevalent conditions associated with HFpEF, namely obesity, impaired glucose tolerance, hyperlipidemia resulting from a Western diet, and hypertension. The Western lifestyle has consistently been linked to the prevalence of cardiovascular diseases. Excessive consumption of sodium and fats are believed to be the primary dietary factors driving this connection. Moreover, in Western nations like the United States, the average consumption of total fiber falls notably below recommended levels (15) with limited information regarding the intake of fermentable or prebiotic fiber. Thus, fiber is also recognized as a crucial element in preventing cardiovascular disease (16). In fact, prior research indicates that lack of fermentable fiber leads to cardiovascular disease in a mouse model of angiotensin II (ANG II) infusion (17).

Currently, there are only a limited number of mouse models that exhibit a HFpEF cardiometabolic phenotype. One combines the *db/db* mouse model with a 4-wk aldosterone infusion, offering a more accurate representation of the diabetic HFpEF clinical phenotype subgroup (18). Another model uses high-fat-fed mice treated with the endothelial nitric oxide synthase (eNOS) inhibitor l-NAME (Nω-Nitro-l-arginine methyl ester) to induce endothelial dysfunction-based hypertension, a widely used approach (19). In our model, rather than directly inhibiting eNOS to induce hypertension, we aimed to explore the impact of metabolic and excess mineralocorticoid-salt challenges on eNOS signaling. This approach was chosen to create a model that naturally develops a downregulation of eNOS signaling, which is crucial in HFpEF pathology, affecting microvascular endothelial function and NO availability for cardiomyocytes (20, 21). Our model recapitulates key features of human HFpEF, including diastolic dysfunction, metabolic disturbances, inflammation, oxidative stress, and alterations in calcium signaling and myofilament proteins. This multichallenge approach resulted in the development of cardiac abnormalities as early as 3 wk after challenge, prompting our investigation into the early pathological mechanisms underlying the HFpEF syndrome.

## MATERIALS AND METHODS

### Animals

All protocols were approved by the Animal Care and Use Committee of the University of Illinois at Chicago, following the approved animal protocol #22–207. C57BL6/J mice, aged 8–10 wk and of both sexes, were obtained from Jackson Laboratory. The mice were housed under standard conditions of temperature, humidity, and lighting. Control mice received regular water and a standard chow diet (Teklad, LM-485) containing 5.8% fat by weight and 13.7% of neutral detergent fiber, including cellulose, hemicellulose and lignin (Supplemental Table S1). A treatment group of mice underwent subcutaneous implantation of controlled-release DOCA pellets at a dosage of 0.83 mg/day (Deoxycorticosterone Acetate, Innovative Research of America, SM-121). These mice also received drinking water supplemented with 1% NaCl and were fed a high-fat low-fiber diet (HFD, Research Diets Inc., D12492) containing 35% fat by weight (60% by calories) and 6.5% of cellulose, for durations of 3 and 6 wk. Mice had ad libitum access to food and water. Neither food consumption nor daily fecal output was monitored. In the following, we will use the term “DOCA + Western diet” model to describe the treatment and the resulting phenotype. The method of euthanasia was cardiac excision performed under isoflurane anesthesia.

### Echocardiography

Mice were anesthetized with isoflurane in an induction chamber, transferred to the Vevo imaging station warming platform and administered isoflurane via a nose cone connected to a vaporizer to deliver 1.5–2.0% isoflurane driven by 100% oxygen (1 L/min). Mice were positioned in the dorsal decubitus position, and chest hair was removed using a depilating agent. Throughout the procedure, body temperature (maintained at 37 ± 0.5°C) and heart rate were monitored. We used Vevo 2100 echocardiography system to capture multiple image views, including B-mode and M-mode images in parasternal long (PLAX) and short axis (PSAX) to measure structural and functional parameters (22–25). Relative wall thickness (RWT), an index of hypertrophy, was calculated using diastolic chamber dimensions in PSAX as follows: (posterior wall + interventricular septum at end-diastole)/(left ventricular internal diameter at end-diastole) (24, 26). The apical four-chamber view was used to evaluate mitral valve flow patterns using pulse wave (PW) Doppler. Tissue Doppler imaging was performed at the septal part of the mitral annulus, as previously described (22–25). A modified PSAX view of the left ventricle was used to assess the pulmonary artery flow by PW Doppler to measure the pulmonary flow velocities, pulmonary acceleration time (PAT), pulmonary ejection time (PET), and cardiac cycle length (CL) (24). Subsequently, the PAT/PET and PAT/CL ratios were calculated based on these values. Coronary flow in the left anterior descending (LAD) coronary artery was examined using a modified PLAX view, as described previously (24). Coronary flow velocity reserve (CFVR) was determined following a stress challenge induced by increasing isoflurane concentration from 1 to 2.5% for 5 min. CFVR was calculated as the ratio of diastolic peak coronary flow velocity (CFV) at maximal flow to diastolic peak CFV at baseline. All measurements and calculations were obtained from three consecutive cycles and analyzed using Vevo Analytic Software (VisualSonics, Toronto, ON, Canada).

### Metabolic Parameters

Body composition, including lean mass, fat mass, and free body fluid, was estimated using NMR (Bruker minispec LF50, Billerica). Glucose metabolism was assessed by measuring fasting blood glucose levels and conducting intraperitoneal glucose tolerance tests using 20% dextrose at a dose of 2 g/kg body weight.

Serum samples were analyzed for total cholesterol, high-density lipoprotein cholesterol (HDL), low-density lipoprotein cholesterol (LDL), and triglycerides using a Beckman Coulter AU480 chemistry analyzer. Total cholesterol measurements (Beckman Coulter, CHOL #OSR6116) involved the hydrolysis of cholesterol esters by cholesterol esterase (CHE), producing free cholesterol. Subsequently, cholesterol oxidase (CHO) converted free cholesterol into cholest-4-en-3-one, generating hydrogen peroxide (H_2_O_2_). The H_2_O2 then reacted with 4-aminoantipyrine and phenol in the presence of peroxidase, forming a red quinoneimine dye, whose absorbance was measured spectrophotometrically at 540/600 nm. For the HDL-Cholesterol test (Beckman Coulter, HDL #OSR6195), free cholesterol in non-HDL-lipoproteins was solubilized and consumed, followed by the release of HDL cholesterol for reaction with cholesterol esterase and a chromogen system, resulting in a blue color complex measured bichromatically at 600/700 nm. Similarly, the LDL-Cholesterol test (Beckman Coulter, LDL #OSR6196) involved solubilizing cholesterol from non-LDL-lipoprotein particles, releasing cholesterol from LDL-lipoproteins, and forming a blue color complex measured bichromatically at 540/660 nm. The triglyceride procedure (Beckman Coulter, TRIG #OSR60118) was based on coupled enzymatic reactions, including hydrolysis of triglycerides, phosphorylation of glycerol, oxidation of glycerol-3-phosphate to produce hydrogen peroxide, and formation of a chromophore read at 660/800 nm, proportional to the triglyceride content.

### Blood Pressure Measurements

Systolic and diastolic blood pressure were noninvasively assessed in conscious mice using the tail-cuff method with the CODA instrument (Kent Scientific). Mice were individually placed in holders on a temperature-controlled platform (37°C), and recordings were conducted. Before testing, mice underwent training to acclimate to brief periods of restraint. Blood pressure was measured over three consecutive days, with readings averaging at least eight measurements per mouse per session.

### C-reactive Protein and IL-6 Analyses

We used the Milliplex mouse cytokine magnetic kit (Millipore, Sigma) to detect IL-6 and the Milliplex Mouse Acute Phase (Millipore, Sigma) to detect C-reactive protein in plasma samples. Blood samples were collected with EDTA according to the manufacturer’s instructions and plasma samples were obtained through centrifugation. The samples were incubated with capture antibodies immobilized on fluorescent-coded magnetic beads followed by the introduction of biotinylated detection antibody and subsequently incubated with Streptavidin-PE conjugate, the reporter molecule, to complete the reaction. The results were quantified based on fluorescent reporter signal using the Luminex analyzer (MAGPIX®).

### Protein Preparation for Western Blots

Ventricular tissue (10–15 mg) was homogenized in standard relaxing buffer (SRB: 75 mM KCl, 10 mM Imidazole pH 7.2, 2 mM MgCl_2_, 2 mM EGTA, 1 mM NaN_3_) at a ratio of 10:1 volume to weight. To all buffers, protease inhibitors [1% (vol/vol); Sigma, P-8340], phosphatase inhibitor cocktail set II [1% (vol/vol); Millipore, 524624], and Calyculin A (100 nM, Cell Signaling Technology, 9902) were added. Using the Bead Ruptor 24 Elite Homogenizer (Omni International, 19-040E), the samples were homogenized at 4°C. After homogenization, the samples were combined with Industrial Sample Buffer (ISB: 8 M urea, 2 M thiourea, 50 mM Tris pH 6.8, 3% SDS, 75 mM DTT, 0.05% bromophenol blue) (27) at a ratio of 5:1 vol/vol and vortexed for 15 min, as previously described (23, 25). The protein concentrations were measured using the Pierce 660 nM Protein Assay with the IDCR reagent (Thermo Fisher Scientific, 22660). Samples were stored at −80°C. Primary antibodies were incubated at 4°C overnight. Antibodies of interest include Thre308 AKT, (1:1,000, Cell Signaling Technology, 4056S), Ser473 AKT (Cell Signaling Technology, 4060S), total AKT (1:1,000, Cell Signaling Technology, 2938S), Ser1176 eNOS (1:1,000, Cell Signaling Technology, 9570S), total eNOS (1:1,000, BD Transduction, N30020), p-VASP (1:1,000, Cell Signaling Technology, 3114), total VASP (1:1,000, Cell Signaling Technology, 3112), NOX2 (gp91phox) (1:1,000, BD Transduction, 61145), PKG-α (1:1,000, Cell Signaling Technology, 13511S), p-CaMKII (1:1,000, Invitrogen, PA5-37833), total CaMKII (1:5,000, Badrilla, A010-56AP), PKA-c (1:1,000, Cell Signaling Technology, 5842S), SERCA2a (1:5,000, Badrilla, A010-23L), GAPDH (1:1,000, Cell Signaling Technology, 2118 L), p-PLB Ser 16 (1:1,000, Millipore, 07–052), and p-PLB Thr 17 (1:2,500, Badrilla, A010-13AP). cMyBP-C phospho-specific rabbit polyclonal antibodies (S273P, S282P, and S302P) were generously provided by Sakthivel Sadayappan, PhD (University of Cincinnati College of Medicine, Cincinnati, OH) and total mouse monoclonal cMyBP-C antibody (1:2,500. Santa Cruz Biotechnology, #SC-137181).

For RyR2 Western blots, ventricular tissue samples underwent a distinct processing method as previously described (28). Tissue samples were mixed with homogenization buffer (0.9% NaCl, Tris-HCl 10 mM pH 6.8, 20 mM NaF, 2 µM leupeptin, 100 µM phenylmethylsulphonyl fluoride, 500 µM benzamidine, 100 nM aprotinin), homogenized using a Teflon pestle, and centrifuged at 1,000 *g* for 5 min at 4°C. Supernatants were aliquoted and stored at −80°C until used. Protein concentrations were determined using the Bradford method (Bio-Rad). Tissue homogenate (50 µg) were suspended in Laemmli buffer and separated by SDS-PAGE in 4–20% TGX precast gels (Bio-Rad). Proteins were then transferred to PVDF membranes using the iblot2 transfer system (ThermoFisher). Membranes were probed with the following antibodies using the ibind flex system (ThermoFisher): anti-RyR [clone 34 C] (1:2,000; MA3-925, ThermoFisher), pS2808-RyR (1:1,000, custom-made) (29), pS2030 (1:1,000, custom-made) (30), pS2814 (1:2,000, A010-31, Badrilla), and GAPDH [clone 6C5] (1:10,000; MAB374, Millipore). Secondary antibodies, used as appropriate, were goat anti-mouse-HRP (1:1,000; 31437, ThermoFisher) or goat anti-rabbit-HRP (1:1,000; 31463, ThermoFisher). Membranes were developed using SuperSignal ECL reagent (ThermoFisher) and imaged with a ChemiDoc MP apparatus (Bio-Rad). Band intensity was quantified with the ImageLab 6.0.1 software (Bio-Rad).

### Myofilament Preparation for Pro-Q Diamond Stain

Mouse ventricles (10–15 mg) were homogenized in standard relaxing buffer as described earlier. To demembranate and purify the myofibrils, Triton-X 100 [1% (vol/vol)] was added to the SRB (SRB-X 100) and incorporated into the sample at 1:10 relative to original tissue weight (31) as previously described (22). Following the procedure, the pellet was resuspended with Industrial Sample Buffer at a 1:5 ratio relative to tissue weight. Protein concentrations were determined using the Pierce 660 nm Protein Assay with the addition of the IDCR reagent (Thermo Fisher Scientific, 22,660). Samples were stored at −80°C. To assess overall myofilament phosphorylation changes, we prepared a 15% SDS-PAGE gel with the following components: 30% acrylamide, 0.5% bis-acrylamide, 1.5 M Tris (pH 8.8), 50% glycerol, 10% SDS, 10% ammonium persulfate (APS), and N, N, N′, N′-Tetramethylethylenediamine (TEMED). The stacking gel consisted of 10% acrylamide, 15% N, N′-Diallyltartardiamide (DATD), 0.5 M Tris (pH 6.8), 50% glycerol, 10% SDS, 10% APS, stacking dye, and TEMED. The gel was run at 200 V for 75 min using Tris-Glycine running buffer (0.025 M Tris base, 0.192 M glycine, 0.1% SDS) in a Criterion cell (Bio-Rad Inc., 1656001, Hercules, CA). After electrophoresis, the gel was stained with Pro-Q Diamond Phosphoprotein Gel Stain (Invitrogen, P33300) to detect phosphorylated proteins, followed by destaining with Pro-Q Diamond Phosphoprotein Gel Destaining Solution (Invitrogen, P33310). To assess total protein content, the gel was then stained with Coomassie G-250 stain (Bio-Rad Inc.). Gel images were captured with ChemiDoc MP (Bio-Rad, Inc.), and band densities were determined and analyzed using ImageLab 6.0.1 software and Microsoft Excel.

### In Gel-Based Proteomics Analysis

To verify the identity of the gel bands labeled as titin, we ran a 15% SDS-PAGE gel using control samples and stained it with Coomassie Blue G-250 to facilitate band visualization. This allowed for the precise excision of the top band, which was then subjected to proteomic analysis. The top gel band was cut into 1–2 mm³ pieces and washed three times with 50% acetonitrile (ACN) and 25 mM ammonium bicarbonate (ABC) for 20 min each. Proteins were reduced with 10 mM Dithiothreitol (DTT) in 25 mM ABC, alkylated with 50 mM iodoacetamide, and washed with 25 mM ABC. The gel pieces were then dehydrated in 100% ACN and air-dried. Rehydration was performed in 50 mM ABC containing 10 μg/mL trypsin, followed by overnight incubation at 37°C. Peptides were extracted using 150 μL of 50% ACN with 0.1% formic acid (FA), incubated for 20 min while shaking, and centrifuged at 14,000 *g* for 5 min. This extraction was repeated twice, and supernatants were pooled and dried. After reconstituting in 25 μL of 5% ACN in 0.1% FA, the samples were desalted using a C18 zip-tip, washed with 5% ACN in 0.1% FA, and eluted with 50% ACN, 0.1% FA. The elutes were dried and suspended in 12 μL of 5% ACN, 0.1% FA for LC-MS. Digested peptides (4 μL) were analyzed using Q Exactive HF mass spectrometer coupled with an UltiMate 3000 RSLC nanosystem with a Nanospray Frex Ion Source (Thermo Fisher Scientific). Digested peptides were loaded into a Waters nanoEase M/Z C18 (100 Å, 5 μm, 180 μm × 20 mm) trap column and then a 75 μm × 150 mm Waters BEH C18 (130 A, 1.7 μm, 75 μm × 15 cm) and separated at a flow rate of 300 nL/min. Solvent A was 0.1% FA in water and solvent B was 0.1% FA, 80% ACN in water. The solvent gradient of LC was 5% B in 0–3 min, 8% B in 3.2 min, 8–35% B in 110 min, 35–95% B in 119 min, wash 95% in 129 min, followed by 5% B equilibration until 140 min. Full MS scans (350–1,400 m/z) were acquired at a resolution of 60,000 (at 200 m/z) with an automatic gain control (AGC) target of 3.00E + 06. The 15 most intense peaks, with charge states of 2, 3, 4, and 5, were selected for fragmentation in the high-energy collision dissociation (HCD) cell with a normalized collision energy of 28%. To prevent repeat fragmentation, these peaks were excluded for 30 s within a mass window of 1.4 m/z. Tandem mass spectra were acquired at a resolution of 30,000, with an AGC target value of 1.00E + 05. The ion selection threshold was set to 1.00E + 04 counts, and the maximum ion injection time was limited to 50 ms for both full and fragment ion scans. For data analyses, spectra were searched against the Uniprot mouse database using MaxQuant (2.0.3.1) with the following parameters: precursor mass tolerance of 10 ppm, fixed modification carbamidomethylation (C), variable modifications oxidation (M), deamidation (NQ), and acetylation (n). Search results were entered into Scaffold DDA software (v6.0.1, Proteome Software, Portland, OR) for compilation, normalization, and comparison of spectral counts, etc.

### Calcium Imaging

To quantify cellular calcium (Ca^2+^)-handling properties, ventricular myocytes were isolated from male and female control and challenged hearts as previously described (32). To isolate cells, hearts were excised, the aorta cannulated, connected to a Langendorff apparatus, and perfused with Ca^2+^-free tyrode solution. The digestion solution was supplemented with 2,3-Butanedione monoxime (0.5 µmol/L), CaCl_2_ (12.7 µmol/L), Liberase blendzyme (60 µg/mL), trypsin (0.014%), and phenol red (0.5%). When tissue started to appear translucent, ventricles were placed into protease (1 mg/mL) solution. The digestion was stopped by addition of bovine calf serum (Hyclone) before extracellular Ca^2+^ was raised progressively to 1 mmol/L. For the measurement of intracellular calcium ([Ca^2+^]_i_), ventricular myocytes were loaded with the membrane-permeable fluorescent calcium indicator Fluo-4/AM (#F14201, Invitrogen). Cells were incubated with the indicator for 15 min and another 15 min were allowed for deesterification after washing with standard Tyrode solution. Fluo-4 loaded cells were excited at 494 nm and the emission signal collected at 506 nm. The signals are presented as background subtracted changes in fluorescence that were normalized to the baseline fluorescence at the beginning of the experiment. Cells were field stimulated at 0.5 Hz throughout the experiment.

### Quantification of Reactive Oxygen Species

Reactive oxygen species (ROS) production was quantified in isolated ventricular myocytes from male and female control and challenged hearts as previously described (32). To quantify the production of ROS, isolated ventricular myocytes were loaded with 2′,7′-dichlorofluorescein diacetate (DCFH) (10 μmol/L for 30 min at 37°C) as previously described (32). DCF fluorescence was monitored (excitation: 494 nm, emission: 506 nm) every 2 min for a period of 18 min. The background subtracted fluorescent signal was normalized to the fluorescence at the onset of the experiment (F_0_) and quantified as the change in fluorescence over time.

### Colon Harvesting and Processing for Histology

After harvesting the colon, its length and weight were measured. Segments of colon, along with digesta and without washing, were carefully collected at a length of 5 mm. Subsequently, the fecal content of the colon was measured. Colon samples were immersed in Carnoy’s fixative and left to fix for a minimum of 3 h. After fixation, the tissues underwent three washes in methanol before being processed and embedded in paraffin. Using an Epredia HM 325 rotary microtome (Thermo-Fisher Scientific, 902100 A), the tissues were sectioned into 5-μm-thin slices, with 4–5 sections per mouse collected and placed on a glass slide. The embedded and sectioned material were stored at room temperature.

### Colon Crypt Length Measurement

Slides were baked for 2 to 3 h at 58°C to melt the paraffin. The slides were cooled down at room temperature and stained with hematoxylin and eosin (H&E) following the kit protocol (StatLab, KTHNEPT). H&E images were scanned using the Aperio Whole Slide Scanning Bright-field ×20 magnification. Four to five images per mouse colon were taken using Aperio ImageScope (v12.4.6.5003) and 15–30 crypts per image were collected. Crypt length measurement was calculated using ImageJ software.

### General Lipopolysaccharide ELISA

Blood samples were collected using EDTA and plasma samples were obtained through centrifugation. Plasma samples were then diluted 10-fold. LPS levels in the plasma samples were measured using the general LPS ELISA Kit (MBS452438, MyBioSource), following the manufacturer’s instructions. This assay uses a competitive inhibition enzyme immunoassay technique where a monoclonal antibody specific to LPS is precoated onto a microplate. The reaction is visualized by adding a substrate solution, and the color intensity developed is inversely proportional to the LPS concentration. Absorbance was measured at 450 nm using a Cytation 5 imaging reader (BioTek), and LPS concentrations were calculated based on an LPS standard curve.

### Colon Mucus Thickness Measurement

Slides were deparaffinized for 3 to 4 min on heater at 60°C. The slides were cooled down at room temperature before placing in Histo Clear (D-Limonene) (National Diagnostics, HS-200) to remove the excessive paraffin followed by rehydration with ethanol (100%, 95%, and 70%, 5 min each). The slides were washed with water and then stained according to the standard Periodic acid-Schiff (PAS) kit (Fisher Chemical, SP15-100). Images were obtained using the Aperio Whole Slide Scanning Bright-field at ×40 magnification. The mucus thickness was measured as the vertical distance between the cell surface and the luminal mucus surface, using imageJ software. Four to five images per mouse colon were taken using Aperio ImageScope (v12.4.6.5003) and 25–50 measurements per image were collected.

### Statistical Analyses

We used GraphPad Prism 10.2.2 Software (GraphPad, Inc.) for statistical analyses. The Kolmogorov–Smirnov test was used to evaluate normality, while Spearman’s rank correlation test was used to detect heteroscedasticity. A one-way ANOVA followed by Tukey’s multiple comparisons test was used to identify significant differences among three groups. The Student’s *t* test was used for comparing two independent groups. In cases of a nonparametric distribution, the Mann–Whitney test and Kruskal–Wallis ANOVA were used for hypothesis testing. A *P* value of less than 0.05 was considered statistically significant. Data were presented as mean ± SE. Male and female data were combined as no statistical differences were observed between sexes, unless stated otherwise in the figures and figure legends.

## RESULTS

### Cardiac Phenotype of Mice following 3 and 6 Wk of Exposure to DOCA + Western Diet

**Figure 1.**
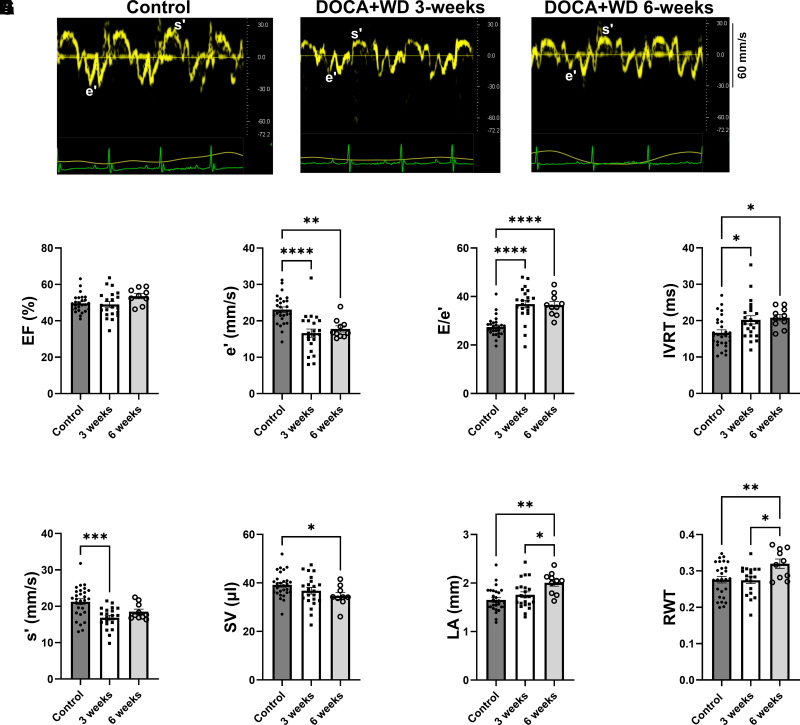
Diastolic dysfunction with preserved ejection fraction after 3- and 6-wk exposure to excess mineralocorticoid and a Western diet. C57BL/6J control mice were given a chow diet. The DOCA+WD group denotes mice subjected to DOCA, high-fat diet (HFD), and saline challenges for 3 and 6 wk. *A*: representative tissue Doppler echocardiographic images 3 and 6 wk after challenge showing peak myocardial relaxation velocity at early diastole (e’) and peak myocardial contraction velocity (s’). *B*: ejection fraction (EF). *C*: peak myocardial relaxation velocity (e’). *D*: ratio between mitral *E* wave and e’. *E*: isovolumetric relaxation time (IVRT). *F*: peak myocardial contraction velocity (s’). *G*: stroke volume (SV). *H*: left atria (LA) diameter. *I*: relative wall thickness (RWT = posterior wall + interventricular septum at end-diastole)/left ventricular internal diameter at end-diastole). Data from both male and female mice were presented together. *n* = 10–28. Data presented as means ± SE. Data analyzed with ANOVA *P < 0.05; **P < 0.01; ***P < 0.001; ****P < 0.0001.

Echocardiographic assessments were performed at 3- and 6-wk postchallenge. While ejection fraction (EF) remained preserved throughout the study period (Fig. 1*B*), mice exposed to the challenge exhibited signs of diastolic dysfunction, including reduced peak myocardial relaxation velocity at the mitral annulus (e') (Fig. 1, *A* and *C*), elevated E/e' ratio (Fig. 1*D*), and prolonged isovolumetric relaxation time (IVRT) (Fig. 1*E*). Contractility parameters, such as peak myocardial contraction velocity at the mitral annulus (s'), showed a transient decline in challenged mice at the 3-wk mark (Fig. 1, *A* and *F*). Moreover, we noted a reduction in stroke volume (SV) (Fig. 1*G*) and cardiac output (CO) (Supplemental Table S2) accompanied by left atrial (LA) enlargement (Fig. 1*H*), a characteristic often observed in individuals with HFpEF. In addition, there was an increase in relative wall thickness (RWT) (Fig. 1*I*), an index of hypertrophy (24, 26), along with an increase in systolic and diastolic blood pressure (Supplemental Table S2). These results suggest that excess mineralocorticoid, salt, and a high-fat, low-fiber diet collectively induce increased arterial blood pressure, increased left ventricular (LV) filling pressure, and diastolic dysfunction, with a concurrent decrease in stroke volume. Data for male and females were combined as no significant differences were found between sexes.

**Figure 2.**
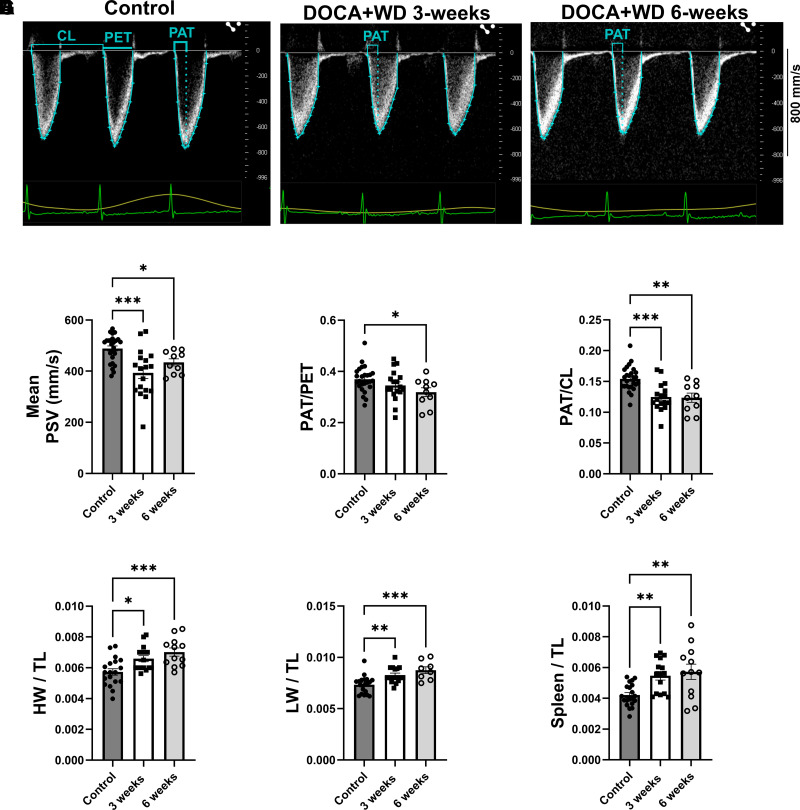
Signs of elevated pulmonary pressure and organ congestion following 3 and 6 wk of exposure to excess mineralocorticoid and a Western diet. Control C57BL/6J mice were assigned to a standard chow diet. The group labeled as DOCA+WD indicates mice exposed to DOCA, high-fat diet (HFD), and saline challenges for 3 and 6 wk. *A*: representative flow Doppler echocardiographic images of pulmonary systolic velocity (PSV). Pulmonary acceleration time (PAT) represents the duration from the onset of acceleration to the attainment of peak velocity. Pulmonary ejection time (PET) is assessed from the onset of acceleration until the return to baseline. Cardiac cycle length (CL) denotes the interval from the initiation of acceleration in one cycle to the initiation of acceleration in the subsequent cycle. *B*: mean PSV. *C*: PAT/PET ratio. *D*: PAT/CL ratio. *E*: ratio of heart weight (HW, g) and tibia length (TL, mm). *F*: ratio of lung weight (LW, g) and tibia length (TL, mm). *G*: ratio of spleen weight (g) and tibia length (TL, mm). Data from both male and female mice were presented together. *n* = 8–26. Data presented as means ± SE. Data analyzed with ANOVA *P < 0.05; **P < 0.01; ***P < 0.001.

Following the 3- and 6-wk challenge, we observed signs of elevated pulmonary pressure in mice exposed to DOCA + Western diet. These changes included a decrease in mean pulmonary systolic velocities (PSVs) (Fig. 2, *A* and *B*), alongside the peak velocity occurring earlier in systole. This resulted in a shortened pulmonary acceleration time (PAT) and smaller ratios of PAT to pulmonary ejection time (PET) (Fig. 2*C*) and PAT to cycle length (CL) (Fig. 2*D*). Moreover, increased organ weights were observed in the heart (Fig. 2*E*), lungs (Fig. 2*F*), and spleen (Fig. 2*G*), suggesting cardiac hypertrophy and organ congestion, which are indicative of preclinical signs of heart failure. With cardiac dysfunction established by the 3-wk interval, our focus turned to exploring the initial pathological mechanisms driving cardiac dysfunction in this model. Our aim was to pinpoint the earliest events that could be potentially modified to halt disease progression. Subsequently, all further measurements focused on this initial 3-wk challenge period.

### Initial Metabolic Manifestations Resulting from a Combination of Excess Mineralocorticoid + Western Diet

**Figure 3.**
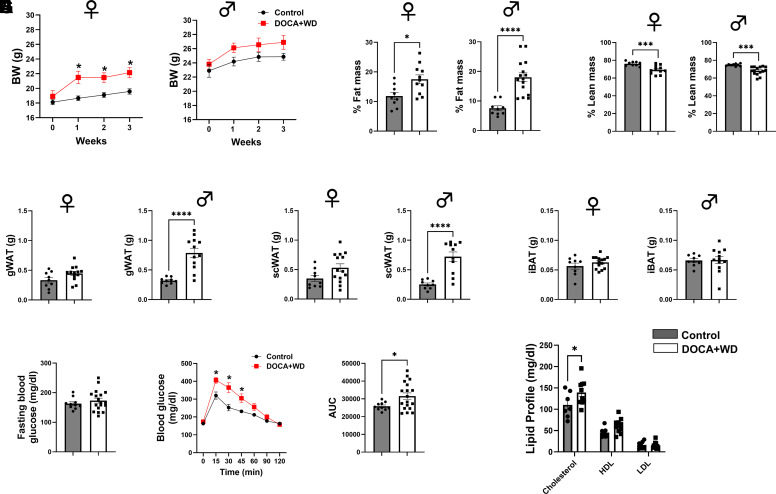
Metabolic phenotype of mice after 3 wk of exposure to excess mineralocorticoid and a Western diet. Control C57BL/6J mice were given a standard chow diet, while the DOCA+WD group signifies mice exposed to DOCA, high-fat diet (HFD), and saline challenges for 3 wk. *A*: weekly body weight (BW) progression of female and male mice. *B* and *C*: % fat mass and % lean mass measured by NMR of female and male mice, separately. Gonadal white adipose tissue (gWAT) (g) (*D*) and subcutaneous (sc) WAT weights (g) (*E*) of female and male mice, separately. *F*: interscapular brown adipose tissue (iBAT) weight (g) of female and male mice, separately. *G*: fasting blood glucose levels (mg/dL), intraperitoneal glucose tolerance test (GTT), and area under the curve (AUC) of the GTT of male and female mice together. *H*: total cholesterol, high-density lipoprotein (HDL) and low-density lipoprotein (LDL) cholesterol of male and female mice together (mg/dL). *n* = 9–18. Data presented as means ± SE. *A–G* analyzed with Student’s *t* test. *H* analyzed with two-way ANOVA *P < 0.05; ***P < 0.001; ****P < 0.0001.

As expected, the treatment group that received a Western diet exhibited a notable increase in body weight, particularly pronounced in females when compared with their controls (Fig. 3*A*). We also observed elevated fat mass (Fig. 3*B*) and reduced lean mass (Fig. 3*C*), assessed using NMR. Furthermore, an increase in visceral adiposity was noted, primarily attributed to increased accumulation of white adipose tissue (WAT) including gonadal (g) depots (Fig. 3*D*) and subcutaneous (sc) depots (Fig. 3*E*), especially in males, with no differences in interscapular (i) brown adipose tissue (BAT) (Fig. 3*F*). Intraperitoneal glucose tolerance test demonstrated impaired glucose tolerance (Fig. 3*G*). In addition, hypercholesterolemia (Fig. 3*H*) was noticeable.

### Exposure to Excess Mineralocorticoid and a Western Diet for 3 Wk Lead to Inflammation, Increased Reactive Oxygen Species, Impaired NO Signaling, and Alterations in Coronary Flow Reserve

**Figure 4.**
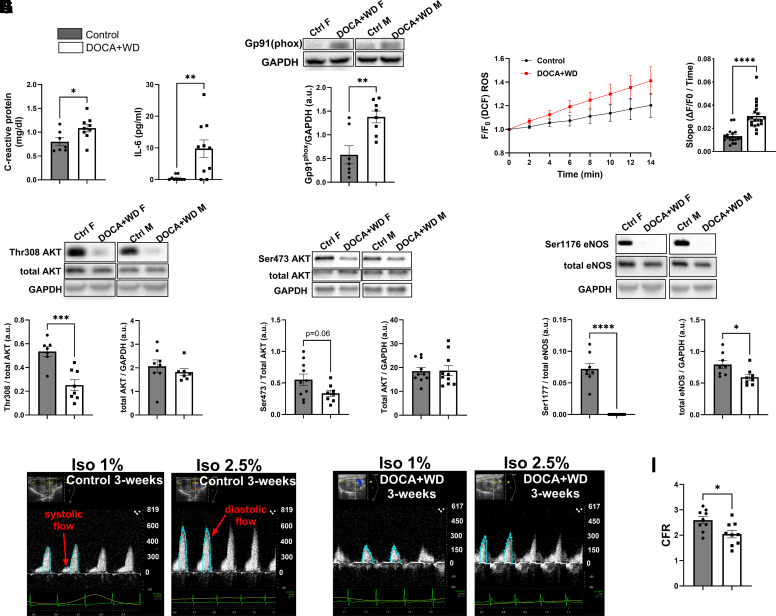
Inflammation, oxidative stress, downregulation of nitric oxide signaling, and impaired coronary flow reserve after 3 wk of exposure to excess mineralocorticoid and a Western diet. C57BL/6J control mice were fed a standard chow diet for 3 wk, while the DOCA+WD group indicates mice exposed to DOCA, high-fat diet (HFD), and salt challenges for the same duration. *A*: C-reactive protein and interleukin-6 (IL-6) detected by ELISA. *B*: Western blot representative images of Gp91(phox) regulatory subunit of nicotinamide dinucleotide phosphate (NADPH) oxidase in females (*left*) and males (*right*) from whole heart homogenates, and summarized quantification of Gp91(phox) abundance over GAPDH. *C*: quantification of the production of reactive oxygen species (ROS) in isolated ventricular myocytes using 2′,7′ dichlorofluorescein diacetate (DCFH). *D*: representative Western blot images of females (*left*) and males (*right*) from whole heart homogenates, alongside summarized quantification of phospho-specific antibodies targeting AKT threonine 308 and AKT serine 473 over total AKT (*E*), including summary data for AKT abundance over GAPDH. *F*: Western blot images representing females (*left*) and males (*right*) from whole heart homogenates, along with summarized quantification of phospho-specific antibodies to endothelial nitric oxide synthase (eNOS) at serine 1177 over total eNOS and summary data for eNOS abundance over GAPDH. *G*: representative pulse-wave Doppler tracings of coronary flow under basal conditions (Isoflurane, Iso 1%) and following hyperemic stimulus (Isoflurane, Iso 2.5%) in controls and challenged mice (*H*). *I*: coronary flow velocity reserve (CFVR) quantification using peak coronary flow velocity values. Data from both female and male mice were combined and presented together. *n* = 7–10. Data presented as means ± SE. Data analyzed with Student’s *t* test. *P < 0.05; **P < 0.01; ***P < 0.001; ****P < 0.0001.

In our model, the simultaneous exposure to DOCA, high-salt and a high-fat low-fiber diet for 3 wk induced a systemic low-inflammatory state, as indicated by elevated levels of C-reactive protein, and IL-6 in the plasma (Fig. 4*A*). As previously stated, the prevailing model in HFpEF pathophysiology suggests that comorbidities such as obesity, diabetes, and hypertension induce a systemic proinflammatory state, contributing to cardiac remodeling through microvascular endothelial dysfunction (20). At the molecular level, we observed elevated markers of oxidative stress in the challenged mice, as evidenced by increased NOX2 (Gp91phox subunit) abundance in ventricular tissue (Fig. 4*B*) and an increased production of intracellular reactive oxygen species (ROS) in isolated ventricular cardiomyocytes (Fig. 4*C*) from challenged mice compared with controls. These changes were accompanied by a notable decrease in AKT phosphorylation, a key upstream regulator of eNOS, at threonine 308 (Fig. 4*D*), and a trend toward a decrease at serine 473 (Fig. 4*E*). The decrease in AKT phosphorylation coincided with a significant reduction in eNOS phosphorylation and eNOS abundance (Fig. 4*F*), suggesting a decline in nitric oxide (NO) availability. Moreover, challenged mice displayed coronary artery dysfunction, as evidenced by a reduction in coronary flow velocity reserve (CFVR) as early as 3 wk into the intervention (Fig. 4, *G* and *H*, Supplemental Table S5). This reduction in CFVR was determined by the ratio of diastolic peak coronary flow velocity (CFV) during maximal hyperemic flow (isoflurane 2.5%) to diastolic peak CFV at baseline (isoflurane 1%) (Fig. 4*I*).

### Dysregulation of Calcium-Handling Proteins and Disrupted Calcium Kinetics Accompany Cardiac Dysfunction after 3 Wk of Challenge

**Figure 5.**
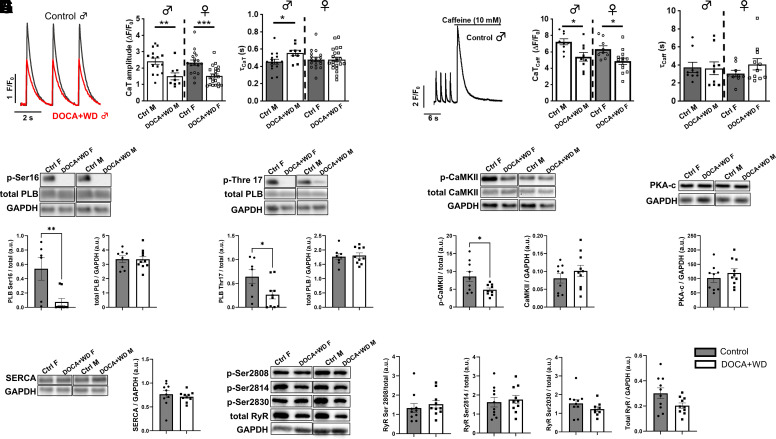
Dysregulation in calcium regulatory proteins and calcium-handling properties following a 3-wk challenge. Control C57BL/6J mice were fed a standard chow diet, whereas the DOCA+WD group consisted of mice exposed to challenges involving DOCA, a high-fat diet (HFD), and saline during a 3-wk period. *A*: representative plots showing ΔF/F0 obtained from isolated ventricular cardiomyocytes (*left*), calcium transient (CaT) amplitude (*middle*), and the decay constant of CaT, tau (τ_CaT_) (*right*). *B*: representative plots illustrating ΔF/F0 obtained from isolated ventricular cardiomyocytes post-caffeine treatment (*left*), caffeine-induced calcium transients (CaT_caff_) amplitude (*middle*), and the decay constant of the caffeine transient (τ_Caff_) (*right*). *C*: representative Western blot images in females (*left*) and males (*right*) and summarized quantification of phosphorylation of phospholamban (PLB) at serine 16 and threonine 17 over total PLB and total PLB abundance over GAPDH in whole heart homogenates (*D*). *E–G*: representative Western blot images in females (*left*) and males (*right*) and summarized quantification of the phosphorylation and abundance of CaMKII, PKA-c (catalytic subunit), and SERCA over GADPH in whole heart homogenates. *H*: representative images and quantification of phosphorylation of ryanodine (RyR) at serine 2808, 2814, 2830 over total RyR and total RyR abundance over GAPDH. Data from both female and male mice were combined and presented together (*C–H*, *n* = 7–10), except in *A* and *B* where females and males were analyzed separately (*n* = 9–22). Data presented as means ± SE. Data analyzed with Student’s *t* test. *P < 0.05; **P < 0.01; ***P < 0.001.

**Figure 6.**
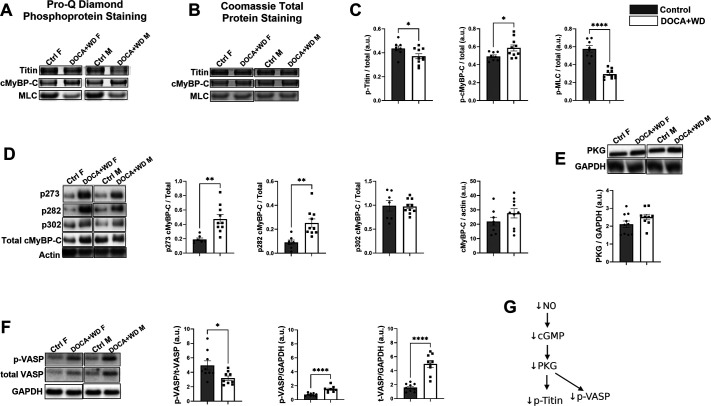
Exposure to excessive mineralocorticoids and a Western diet for 3 wk affected myofilament proteins phosphorylation. Control C57BL/6J mice were provided with a standard chow diet for 3 wk, while the DOCA+WD group refers to mice subjected to DOCA, high-fat diet (HFD), and saline challenges over the same period. Representative images of ProQ diamond-stained SDS-PAGE gel of phosphorylated myofilament proteins (*A*) and Coomassie R-250-stained gel (*B*). *C*: summarized analysis showing the phosphorylation of titin, cardiac myosin binding protein-C (cMyBP-C), and myosin light chain 2 (MLC2). *D*: representative images in females (*left*) and males (*right*) and Western blotting quantification of cMyBP-C at Serine 273, 282, 302 over total cMyBP-C, and total cMyBP-C quantification over actin. *E*: representative Western blot images in females (*left*) and males (*right*) and quantification of PKG over GAPDH. *F*: representative images in females (*left*) and males (*right*) of vasodilator-stimulated phosphoprotein (VASP) phosphorylation, VASP abundance, and GAPDH. Histograms displaying the quantification of VASP phosphorylation relative to VASP abundance, as well as VASP phosphorylation and VASP abundance relative to GAPDH, are shown with adjusted *y*-axis scales. These adjustments were made to improve visualization and facilitate comparisons, particularly emphasizing the reduced phosphorylation of the more abundant VASP protein in the DOCA+WD group. *G*: NO-cGMP-PKG pathway impacts the phosphorylation of titin and VASP. Data from both male and female mice were analyzed together. *n* = 7–9. Data presented as means ± SE. Data analyzed with Student’s *t* test. *P < 0.05; **P < 0.01; ****P < 0.0001.

To determine the cellular calcium handling in our model, isolated ventricular myocytes were loaded with Fluo-4/AM and calcium transients (CaT) were recorded during field stimulation (0.5 Hz). CaT in ventricular myocytes from male- and female-treated mice exhibited a reduced CaT amplitude compared with the respective control cells that coincided with a prolonged CaT decay constant (τ) only in males (Fig. 5*A*). Depletion of the sarcoplasmic reticulum (SR) by caffeine (10 mM) revealed a reduced SR load in male- and female-challenged ventricular myocytes (Fig. 5*B*). The decay of the caffeine transient was not altered suggesting that there is no significant change in NCX activity, but the prolonged CaT decay is a consequence of attenuated SERCA activity. Consistent with this, we noted a decrease in phospholamban phosphorylation at serine 16 (Fig. 5*C*) and threonine 17 sites (Fig. 5*D*) in both female and male mice exposed to excess mineralocorticoid and a Western diet. These changes in phospholamban were accompanied by decreased CaMKII phosphorylation (Fig. 5*E*), responsible for phosphorylating threonine 17, but no differences were detected in PKA-c (Fig. 5*F*) or PKG expression (Fig. 6*E*), which phosphorylate phospholamban at serine 16. In addition, there were no changes observed in cardiac sarcoplasmic reticulum Ca^2+^-ATPase (SERCA2) (Fig. 5*G*) or in the phosphorylation and abundance of ryanodine receptors (RyRs) (Fig. 5*H*).

### Exposure to Excess Mineralocorticoid and a Western Diet for 3 Wk Affects the Phosphorylation of Myofilament Proteins

Using a 15% SDS-PAGE gel, we assessed the phosphorylation status of myofilament proteins with Pro-Q Diamond staining (Fig. 6*A*) and estimated total protein levels with Coomassie staining (Fig. 6*B*, Supplemental Fig. S11). Our analysis revealed a decrease in the phosphorylation of titin and myosin light chain-2 (MLC2). This was accompanied by elevated levels of cardiac myosin binding protein-C (cMyBP-C) phosphorylation (Fig. 6*C*), with no changes detected in the phosphorylation of other myofilament proteins (data not shown). Then, we used Western blots to assess the phosphorylation levels of cMyBP-C at its three key sites: serine 273, 282, and 308. In line with the findings from proQ Diamond staining, we observed a significant increase in phosphorylation at serine 273 and 282 in challenged mice (Fig. 6*D*). This rise in cMyBP-C phosphorylation could potentially serve as a compensatory mechanism to offset reduced CaT amplitude and SR load, thereby ensuring the maintenance of systolic function and the preservation of ejection fraction.

Although the 15% SDS-PAGE gel used in our analysis is not optimal for specifically detecting titin, it allows for the visualization of a wider range of myofilament proteins and their molecular weights compared with SDS-VAGE (vertical agarose gel electrophoresis) (33). Given the limitations of this method for titin detection, we sought to confirm its identity using proteomic analysis. To this end, we prepared an additional 15% SDS-PAGE gel and loaded it with samples of control myofilament preparations under identical conditions. After electrophoresis, we carefully excised the top protein bands corresponding to those labeled as titin in Fig. 6*B* and processed them following the protocol outlined in materials and methods. Subsequent proteomic analysis identified titin as the top hit, achieving ∼55% sequence coverage with a ≤ 1% false discovery rate (FDR), thereby verifying its presence in the gel bands labeled as titin (see Supplemental File 2). To investigate the molecular mechanism driving the decrease in titin phosphorylation, our attention turned to PKG signaling, a kinase responsible for titin phosphorylation. We examined the abundance of PKG, which showed no significant difference compared with the control (Fig. 6*E*). Following this, we redirected our attention to other proteins that undergo phosphorylation via cGMP-PKG signaling, including vasodilator-stimulated phosphoprotein (VASP). VASP is phosphorylated by PKG at serine 239 and has previously been identified as a sensitive indicator of impaired NO-cGMP signaling and endothelial dysfunction (34). Our findings revealed a significant decrease in VASP phosphorylation in our model, accompanied by an increase in VASP abundance (Fig. 6*F*). This could potentially represent a compensatory mechanism in response to decreased VASP phosphorylation and activity, providing additional support for the downregulation of the NO-cGMP-PKG signaling pathway (Fig. 6*G*).

### Excess Mineralocorticoid and a Western Diet Induced Intestinal Atrophy

**Figure 7.**
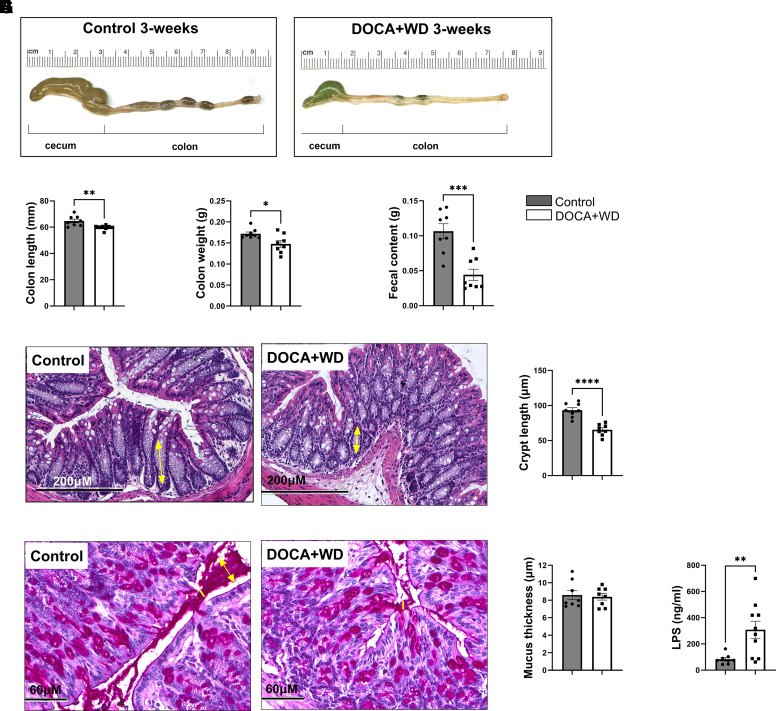
The 3-wk challenge results in significant atrophy in the large intestine. C57BL/6J mice were fed chow or challenged with DOCA + Western diet for 3 wk. *A*: representative images of cecum and colon. *B*: colon length (mm). *C*: colon weight (g). *D*: fecal content (g). *E*: histological images of colon by H&E staining. Scale bars, 200 µm. *F*: colon crypt length, as shown by a double-headed yellow arrow, was measured. *G*: the thickness of the mucus layer, as indicated with yellow lines and double-headed arrow, was measured in samples stained with Periodic Acid-Schiff (PAS). Scale bars, 60 µm. *H*: mucus layer thickness, as shown by double-headed yellow arrow, was measured. *I*: lipopolysaccharide (LPS) levels in plasma (ng/mL). Data from both male and female mice were presented together. *n* = 8. Data presented as means ± SE. Data analyzed with Student’s *t* test. *P < 0.05; **P < 0.01; ***P < 0.001; ****P < 0.0001.

In heart failure, the gut hypothesis suggests that a combination of reduced cardiac output causing decreased perfusion in the splanchnic arteries, combined with elevated gut venous pressure, leads to ischemia (35). This, in turn, impacts the intestinal lining, resulting in atrophy and increased intestinal permeability (36). In our model, mice exposed to excess mineralocorticoid, excess salt, and a diet high in fat and low in fiber for 3 wk showed a notable reduction in colon length (Fig. 7, *A* and *B*) and colon mass (Fig. 7*C*), accompanied by decreased fecal material content in the colon (Fig. 7*D*). Accordingly, we investigated crypt length to identify potential differences and observed decreased crypt length in HFpEF mice (Fig. 7, *E* and *F*), with no significant variations in mucus thickness (Fig. 7, *G* and *H*). Moreover, we observed elevated LPS plasma levels in the challenged mice (Fig. 7*I*), suggesting compromised gut integrity and the translocation of bacterial products to the bloodstream. Taken together, these findings suggest that in our model, the combination of mineralocorticoids, salt, and a diet high in fat but low in fiber, particularly lacking fermentable fiber, led to intestinal atrophy, which could potentially impact intestinal permeability and microbial infiltration exacerbating the proinflammatory state.

## DISCUSSION

Employing a combination of previously used methods, we have developed a novel murine model of HFpEF. This model involves the administration of DOCA, an aldosterone precursor, alongside a Western diet high in fat and salt and low in fiber. This model replicates the diverse features of the HFpEF syndrome, including impaired lusitropy, decreased stroke volume and cardiac output, preserved ejection fraction, and notable extracardiac comorbidities such as organ congestion, signs of elevated pulmonary pressure, increased adiposity, and impaired glucose metabolism. In addition, we have uncovered key pathological mechanisms in the early stages of the disease, including a systemic proinflammatory state, oxidative stress, and decreased eNOS phosphorylation impacting the NO-cGMP-PKG pathway. Furthermore, alterations in calcium-handling proteins and CaT amplitude were observed. We hypothesize that the proinflammatory condition could be exacerbated by intestinal atrophy, leading to the migration of microbial wall products into the bloodstream, as indicated by increased LPS levels in the challenged mice. The proposed mechanisms described in this model offer valuable insights into disease pathology and potential therapeutic targets.

### Preclinical Modeling of HFpEF

Previous studies used a combination of methodologies—DOCA pellets in conjunction with unilateral nephrectomy and sodium chloride administered in water—as established protocols for inducing heart failure (37, 38). Although effective in simulating essential hypertension, this approach’s induction of blood pressure elevation via unilateral nephrectomy does not entirely mirror the clinical presentation in most patients with hypertension in terms of the rate and magnitude of increase. Moreover, the induction of hypertension alone does not replicate the metabolic challenges seen in HFpEF. Another model, known as the SAUNA model, involves 1% saline administration, uninephrectomy, and d-aldosterone infusion for 4 wk. While this model effectively induces ventricular hypertrophy and diastolic dysfunction, it fails to produce a metabolic phenotype, showing only dysfunctional brown adipose tissue (12). These limitations highlight the need for models that better mimic the multifaceted metabolic disturbances observed in HFpEF patients. Recognizing that relying solely on a high fat diet did not lead to heart failure, other studies incorporated l-NAME, which accelerates hypertension driven by endothelial dysfunction by inhibiting eNOS, thereby inducing HFpEF (19). In our study, rather than directly inhibiting eNOS, we aimed to investigate the effects on eNOS following exposure to a high-fat diet, salty water, and excess mineralocorticoids. With this, we sought to emulate the dietary conditions of a Western diet—rich in fats, low in fiber, and high in salt—alongside excess mineralocorticoid (39), which have been associated with adverse outcomes in patients with HFpEF (40). This approach reflects the common comorbidities associated with patients with HFpEF, among whom ∼40–50% present with obesity and another 30–50% with diabetes (41).

Other animal models that mimic the cardiometabolic syndrome observed in HFpEF include the ZSF-1 obese rat that combines the spontaneous hypertensive rat with a Zucker diabetic fat rat model. This model induces hypertension and metabolic syndrome, resulting in increased myocardial stiffness due to titin hypo-phosphorylation (42). Another model explored the effects of metabolic-hypertensive stress induced by ANG II and a HFD in 8-wk-old male and female mice over 28 days. This “two-hit” approach resulted in cardiac hypertrophy, concentric LV remodeling, LA enlargement, and reduced exercise capacity (43). In addition, a separate study used continuous infusion of ANG II and phenylephrine, resulting in exercise intolerance, pulmonary edema, concentric myocardial hypertrophy, diastolic dysfunction, microvascular impairment, and fibrosis (44). To reflect the higher prevalence of HFpEF in women (45), other models use only female subjects. For example, female Landrace pigs on a Western diet rich in salt, fat, cholesterol, and sugar, treated with DOCA (100 mg/kg), exhibited LV hypertrophy and left atrial dilation with preserved ejection fraction and decreased titin phosphorylation over 12 wk (46). Similarly, the Gottingen miniswine model, using young females predisposed to metabolic syndrome and treated with DOCA while on a high-fat, fructose, and salt Western diet, developed HFpEF (47). However, this model also showed depressed systolic myofibrillar function with no differences in resting tension (48).

In the present study, we used both female and male mice and observed some sex differences primarily in body weight, with females gaining more weight than males (Fig. 3*A*). In the l-NAME and HFD study, female mice exhibited a milder phenotype than males, which persisted even after ovariectomy (49), suggesting that low estrogen levels may not significantly contribute to HFpEF pathophysiology. In the same model, female mice were subjected to accelerated ovarian failure (AOF) using vinylcyclohexene dioxide (VCD) to mimic natural menopause. Similarly, AOF did not markedly sensitize female mice to HFpEF; only subclinical cardiac dysfunction was detected through strain imaging. In addition, AOF-HFpEF mice showed increased infiltration of leukocytes in the myocardium (50). In another study where male and female felines were subjected to slow progressive pressure overload via aortic banding, both sexes developed similar cardiopulmonary dysfunction. However, females demonstrated a less pronounced response to pressure overload compared with males (51). Conversely, female *db/db* mice challenged with aldosterone infusion for 4 wk displayed more severe diastolic dysfunction, characterized by an increase in the titin N2B isoform and increased titin phosphorylation in the PEVK region (52).

Through echocardiography, our model displayed diastolic dysfunction, and a reduction in stroke volume and cardiac output, while ejection fraction was maintained (Fig. 1 and Supplemental Table S2). Furthermore, signs of elevated pulmonary pressure and organ congestion were observed (Fig. 2 and Supplemental Table S3). Cardiac dysfunction became apparent at the 3-wk interval, directing our focus toward exploring the initial pathological mechanisms driving cardiac dysfunction in our model.

### Elevated Inflammation and Oxidative Stress Impair the NO-cGMP-PKG Signaling Pathway

In our model, the combination of elevated fat, salt, and excess mineralocorticoids resulted in a state of low-systemic inflammation characterized by elevated levels of IL-6 (53) and C-reactive protein (54) (Fig. 4*A*), previously associated with HFpEF. This inflammatory state was accompanied by an increase in NOX2 levels in the heart (Fig. 4*B*), which may have contributed to the increase in ROS production (Fig. 4*C*). In left ventricular myocardial biopsies from patients with HFpEF, prior research has shown that microvascular inflammation and endothelial activation were linked to elevated levels of NOX2 expression observed in both macrophages and endothelial cells. Several enzyme systems, including NADPH oxidases (NOX), xanthine oxidases, and uncoupled eNOS, have the potential to generate ROS and are particularly significant in this context (55). Increased oxidative stress can result in increased degradation of NO through its reaction with ${\text{O}}_{2}^{-}$, ultimately leading to eNOS uncoupling mediated by ONOO^−^, thus contributing to endothelial dysfunction. In the DOCA, salt, and unilateral nephrectomy model, animals exhibited increased production of vascular ROS. Treatment with the eNOS inhibitor l-NAME significantly reduced this ROS production, indicating a substantial contribution of uncoupled eNOS to oxidative stress in vascular tissue (56). Moreover, in p47phox knockout animals, oxidative stress was reduced following unilateral nephrectomy, DOCA and salt treatment, and levels of ROS could no longer be diminished by l-NAME, further emphasizing the role of NOX in oxidative stress regulation (56). In our model, we observed elevated levels of ROS in isolated cardiomyocytes, underscoring the role of local ROS production in the development of HFpEF.

Impaired NO-cGMP-PKG activity has been linked to increased stiffness of cardiomyocytes in HFpEF (20, 57), attributed to decreased phosphorylation of titin (42, 58, 59). Surprisingly, the DOCA + salt+unilateral nephrectomy mouse model exhibited increased titin phosphorylation after 3 wk of challenge (60). In contrast, our model showed a global reduction in titin phosphorylation (Fig. 6, *A*–*C*), which we believe significantly contributes to increased cardiac stiffness. Similarly, the HFD + l-NAME model showed decreased titin phosphorylation (61), further supporting the link between reduced titin phosphorylation and HFpEF. Cardiomyocyte stiffness can be modulated not only by titin phosphorylation but also by alternative splicing of titin, with the N2B isoform being stiffer and the N2BA isoform more compliant (62). A shift to the stiffer N2B isoform and hypo-phosphorylation of titin have been reported in human HFpEF (6, 63) and animal models (42, 64). Furthermore, specific phosphorylation sites significantly influence titin stiffness modulation, making site-specific information essential for understanding the heart’s pathological state. In HFpEF patients, for example, decreased phosphorylation of titin N2B and increased phosphorylation of titin PEVK region have been related to myocardial stiffness (59).

In our model, we also noted a substantial decrease in eNOS phosphorylation at its critical site Ser 1176 (65, 66) (Fig. 4*F*), primarily due to reduced AKT phosphorylation upstream (Fig. 4, *D* and *E*), indicating diminished AKT activity (67). The consequence of reduced NO availability is increased vascular tone, leading to impaired blood flow and contributing to the progression of heart failure. In a previous study involving female Landrace pigs, metabolic syndrome and HFpEF were induced using DOCA and a Western diet rich in salt, fat, cholesterol, and sugar for 12 wk. Unlike our model, this study did not observe changes in AKT or eNOS phosphorylation. However, a shift toward the stiffer N2B isoform of titin was noted. Consistent with our findings, they also observed a reduction in total titin phosphorylation, attributed to a dysfunctional NO-cGMP-PKG axis due to eNOS uncoupling (46).

Despite the lack of efficacy of many drugs used in treating HFrEF for HFpEF, there are exceptions, such as SGLT2 inhibitors. Empagliflozin reduces mortality and hospitalization in patients with HFpEF, regardless of their diabetes status (6). Consistent with this, SGLT2 inhibitors have demonstrated direct cardiovascular benefits beyond glycemic control (68). However, the specific mechanism underlying their cardioprotective effects remains elusive. Studies suggest that empagliflozin and dapagliflozin enhance vascular function and prevent vascular aging by reducing ROS levels and increasing NO availability (69). Moreover, in a pig model of HFrEF that also exhibited diastolic dysfunction, empagliflozin decreased oxidative stress, improved NO-cGMP-PKG signaling, and enhanced titin phosphorylation leading to better cardiomyocyte relaxation (9). Given the elevated ROS levels, decreased eNOS and titin phosphorylation observed in our model, these mechanisms of SGLT2 inhibitors align well and may contribute to their potential cardioprotective effects.

### Increased Phosphorylation Levels of cMyBP-C Likely Play a Role in Preserving the Ejection Fraction despite the Decreased SR Load

In our model, the combination of a Western diet and excess mineralocorticoids led to a decrease in phospholamban phosphorylation (Fig. 5, *C* and *D*). This aligns with findings from previous studies using DOCA, unilateral nephrectomy, and 1% saline drinking water, where an increase in NOS-dependent superoxide production, diminished NO production, and reduced phosphorylated phospholamban were noted (37). Moreover, recent findings suggest that NO can activate CaMKII in cardiac tissue (70, 71). This is consistent with our model, where decreased eNOS phosphorylation and likely eNOS uncoupling suggest a decrease in NO production resulting in diminished CaMKII activity. Furthermore, our observations of decreased levels of phosphorylated CaMKII (Fig. 5*E*) provide additional evidence supporting its decreased activity in our model. Decrease in phospholamban phosphorylation leads to enhanced inhibition of SERCA, attenuating the reuptake of calcium into the SR of cardiac muscle cells and may contribute to impaired lusitropy (Fig. 1). In mice, 80% of the CaT amplitude relies on Ca release from the SR. In our study, we observed a decreased SR load of challenged mice (Fig. 5*B*), implying that the diminished CaT amplitude (Fig. 5*A*) results from reduced SR load. Despite this reduction, our model did not display any signs of impaired contractility, except for a transient decline in peak myocardial contraction velocity, s’, noted at 3 wk of challenge but normalized by 6 wk of challenge (Fig. 1*G*). This preservation of systolic function and ejection fraction is likely attributed to elevated phosphorylation levels at serine 273 and serine 282 of cMyBP-C (Fig. 6*D*).

Previous research has indicated that phosphorylation of cMyBP-C disrupts its N-terminal domain inhibitory interaction with myosin S2, leading to the release of myosin head domains from the filament backbone and enhancing thick filament activation, thereby improving contractility (72, 73). In contrast to our model, the DOCA + salt+unilateral nephrectomy model demonstrated decreased cMyBP-C phosphorylation at Ser282. Furthermore, this model showed increased S-glutathionylation of MyBP-C, which correlates with diastolic dysfunction (60). Similarly, the HFD + l-NAME model also displayed reduced cMyBP-C phosphorylation at Ser282 (61). However, in a diabetic cardiomyopathy model using leptin receptor-deficient *db/db* mice, cMyBP-C phosphorylation levels were significantly elevated (74), similar to what we observed in our study. The authors proposed that increased cMyBP-C phosphorylation is an early indicator of HFpEF and diastolic dysfunction in diabetic cardiomyopathy, and that cMyBP-C hyperphosphorylation may be linked to the pathogenesis of this condition. In our model, despite initial suggestions of impaired contraction due to decreased SR calcium levels, we believe that the compensatory increase in cMyBP-C phosphorylation likely aids in maintaining or even enhancing contractile function by activating the thick filaments, contributing to preserved ejection fraction. Interestingly, we also observed a significant decrease in the phosphorylation of MLC2 in the challenged mice (Fig. 5, *A*–*C*). The relationship between cMyBP-C levels and decreased MLC2 phosphorylation has been previously observed in a knockout cMyBP-C mouse model, where the absence of cMyBP-C correlated with notably low levels of MLC2 phosphorylation (data not shown). Prior studies have indicated that MLC2 phosphorylation likely disrupts the interaction between the N-terminal domain of cMyBP-C and the MLC2 of folded motors, promoting the formation of cMyBP-C links with actin to enhance thin filament calcium sensitivity and improve contraction (75). However, when phosphorylation levels of cMyBP-C are already elevated, the phosphorylation of MLC2 may decrease as a compensatory mechanism to prevent excessive formation of cMyBP-C links with the thin filament, thereby regulating systole.

### Intestinal Atrophy May Contribute to the Initial Low Systemic Inflammatory State

**Figure 8.**
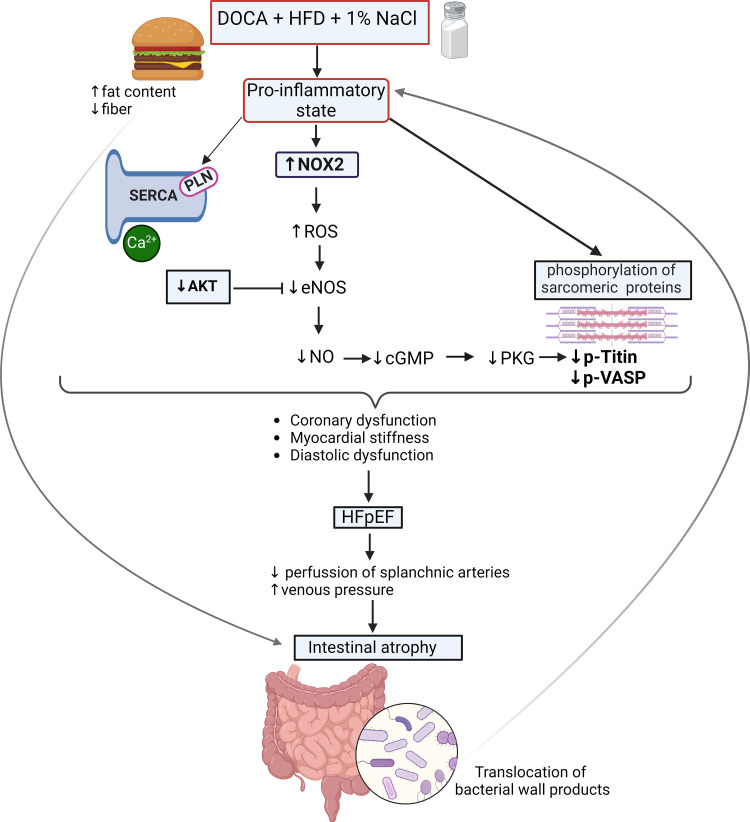
Scheme illustration of the proposed early underlying mechanisms of heart failure after 3 wk of excess mineralocorticoids, high salt intake, and a diet rich in fat and low in fiber. Challenges induce a proinflammatory state, resulting in increased levels of reactive oxidative species (ROS). This elevation in ROS may lead to eNOS uncoupling and a reduction in eNOS activity. This effect is compounded by decreased eNOS phosphorylation through reduced AKT activity. The resultant decline in eNOS activity leads to diminished titin phosphorylation via the downregulation of the NO-cGMP-PKG pathway, contributing to cardiac stiffness and diastolic dysfunction, as indicated by echocardiography. In addition, reduced NO and vasodilator-stimulated phosphoprotein (VASP) phosphorylation may suggest endothelial dysfunction. We also observed decreased coronary flow reserve suggesting coronary dysfunction. There are also alterations in calcium regulatory proteins, such as increased phospholamban-mediated inhibition of SERCA affecting calcium dynamics. These initial pathological mechanisms contribute to heart failure, which can subsequently lead to intestinal hypoperfusion and increased gut venous pressure, resulting in intestinal atrophy, and compromise of intestinal lining integrity. Furthermore, a HFD, particularly one lacking fermentable fiber, can independently induce intestinal atrophy and further compromise gut integrity. These changes may facilitate more direct interactions between the microbiome and host cells, which can then enter the bloodstream as indicated by elevated plasma LPS levels, potentially intensifying the proinflammatory state. Images were created with a licensed version of BioRender.com.

In our model, we noted a marked decrease in colon length, colon mass, and fecal content (Fig. 7, *A*–*D*) together with decreased crypt length (Fig. 7, *E*–*F*). We propose that the simultaneous consumption of a high-fat, high-salt diet along with excess mineralocorticoids results in decreased cardiac output (Supplemental Table S2), leading to reduced gut perfusion, elevated venous pressure, and subsequent intestinal atrophy. However, it is important to note that a HFD alone can contribute to intestinal atrophy and a “leaky gut.” For example, one study demonstrated that HFD-fed mice had reduced expression of proteins like occludin, antimicrobial peptides (Reg IIIβ/γ), and IL-22, leading to increased intestinal permeability and LPS infiltration, suggesting a leaky gut (76). Another study found that HFD induced atrophy in the small intestine, colon, and gut-associated lymphoid tissue (GALT), reducing intraepithelial and lamina propria lymphocytes. The study proposed that this “intestinal lipotoxicity” is driven by the cytotoxicity of free fatty acids derived from the HFD, impairing intestinal immune cells (77). Moreover, the effect of the HFD in our model may be further exacerbated by the absence of fermentable fiber, as has been demonstrated in previous studies (78). However, as our study did not include a group receiving only the HFD, it is challenging to determine whether the observed intestinal atrophy is solely due to the HFD or the combined effects of the high-fat, high-salt diet with excess mineralocorticoids. These interventions ultimately resulted in intestinal atrophy, potentially allowing closer proximity and infiltration of microbiota, followed by translocation into the bloodstream as indicated by elevated LPS levels in the challenged mice (Fig. 7*I*). Previous studies have shown compromised gut integrity and LPS presence in the bloodstream in patients with HF (79). Increased LPS levels initiate immune responses, leading to inflammation, as supported by the higher cytokine levels found in patients with HF, which are associated with symptom severity and prognosis (80, 81). Accordingly, in our model, we observed elevated levels of IL-6 and C-reactive protein (Fig. 4*A*). This may create a detrimental cycle of gut barrier dysfunction and systemic inflammation, leading to oxidative stress in cardiac tissue as indicated by the increased ROS production by cardiomyocytes (Fig. 4*C*). This ongoing process perpetuates the initial inflammation and oxidative stress, thereby creating a continuous loop that worsens the overall cardiac condition (Fig. 8).

### Conclusions

In summary, our findings indicate that combining DOCA-salt-induced hypertension with a Western diet high in fat and salt, and low in fiber, effectively replicates key features of the HFpEF syndrome. Through this model, we have revealed significant pathological mechanisms in the early stages of the disease, including systemic proinflammatory states, oxidative stress, and decreased eNOS phosphorylation that affects the NO-cGMP-PKG pathway. Further findings include alterations in calcium handling and myofilament proteins, along with intestinal atrophy, which may intensify systemic inflammation. These findings offer valuable insights into HFpEF pathology, highlighting the importance of addressing dietary factors and inflammatory processes in the management of this prevalent and challenging cardiovascular condition.

### Limitations

In our model, we detected a global reduction in titin phosphorylation, which we believe plays a significant role in increased cardiac stiffness. However, we did not measure myocardial stiffness directly. In addition, we did not examine phosphorylation at specific regions of titin, such as the N2B or PEVK regions, nor did we assess the relative expression of the more compliant N2BA isoform compared with the stiffer N2B titin isoform. The latter is an important consideration, as an increased expression of the stiffer N2B isoform has been identified as a significant factor in human HFpEF (82), and emerging research is focused on promoting this shift in titin splicing toward a more compliant N2BA isoform (83). Addressing these gaps could provide a more comprehensive understanding of the molecular mechanisms underlying changes in cardiac function.

In this study, we used young mice (8–10 wk old) and observed the development of the phenotype within 6 wk, with cardiac dysfunction and underlying pathologies becoming evident as early as 3 wk. Other models, such as the HFD and l-NAME model, develop the HFpEF phenotype in young mice within 5 wk. In addition, the *db/db* mouse model challenged with aldosterone infusion develops the HFpEF phenotype in just 4 wk using young mice. These studies, including ours, primarily use young adult mice and observe the HFpEF phenotype within a relatively short period. It is crucial to acknowledge that this approach does not fully replicate the HFpEF condition, which predominantly affects older populations and takes years to develop in humans. The exclusion of older age and a more prolonged challenge in our study are significant limitations.

Furthermore, HFpEF is more prevalent in women, particularly after menopause. Although we included both sexes in this study, very few sex differences were found after the 3-wk challenge. The differences observed were mainly in the metabolic phenotype, with females showing more body weight gain than males, similar to previous studies (84, 85). It is possible that after longer periods of challenge, cardiometabolic phenotypes and the underlying molecular mechanisms will become more evident, necessitating further studies.

## DATA AVAILABILITY

Data will be made available upon reasonable request.

## SUPPLEMENTAL MATERIAL

Supplemental Tables S1–S7 and Supplemental Fig. S1–S14: dx.doi.org/10.6084/m9.figshare.26634418.v1.

## GRANTS

This work was funded by National Heart, Lung, and Blood Institute Grants K01HL155241 and K01HL155241-02S1 (to P.C.R.), R01HL161070 and R01HL167195 (to F.J.A.), and R01HL155762 and R01HL164453 (to K.B.); American Heart Association (AHA) Grants CDA849387 and 24IVPHA1299922 and a University of Illinois, College of Pharmacy Vahlteich award (to P.C.R.); and AHA Grant 24IAUST1201236 (via UICHeart: University of Illinois Undergraduate Mentoring and Experience in Heart Research).

## DISCLOSURES

LN is an employee of AbbVie and may own AbbVie stock. None of the other authors has any conflicts of interest, financial or otherwise, to disclose.

## AUTHOR CONTRIBUTIONS

P.C.R. conceived and designed research; P.C.R., L.A.A.N., N.P., D.T., C.H.P., K.R.B., and J.Z. performed experiments; P.C.R., D.T., C.H.P., K.R.B., J.Z., and K.B. analyzed data; P.C.R., L.A.A.N., J.S., K.B., and F.J.A. interpreted results of experiments; P.C.R., D.T., C.H.P., J.Z., and K.B. prepared figures; P.C.R. drafted manuscript; P.C.R., L.A.A.N., and K.B. edited and revised manuscript; P.C.R., L.A.A.N., N.P., D.T., C.H.P., K.R.B., J.Z., J.S., F.J.A., and K.B. approved final version of manuscript.
